# Interlacing Infills for Multi-Material Fused Filament Fabrication Using Layered Depth Material Images

**DOI:** 10.3390/mi13050773

**Published:** 2022-05-14

**Authors:** Irfan Mustafa, Tsz Ho Kwok

**Affiliations:** Department of Mechanical, Industrial and Aerospace Engineering, Concordia University, Montreal, QC H3G 1M8, Canada

**Keywords:** multi-material additive manufacturing, infill, layered depth image, fused filament fabrication, interlacing

## Abstract

One major concern regarding multi-material additive manufacturing (MMAM) is the strength at the interface between materials. Based on the observation of how nature puts materials together, this paper hypothesizes that overlapping and interlacing materials with each other enhance the interface bonding strength. To test this hypothesis, this research develops a new slicing framework that can efficiently identify the multi-material regions and develop interlaced infills. Based on a ray-tracing technology, we develop layered depth material images (LDMI) to process the material information of digital models for toolpath planning. Each sample point in the LDMI has an associated material and geometric properties that are used to recover the material distribution in each slice. With this material distribution, this work generates an interlocking joint and an interlacing infill in the regions with multiple materials. The experiments include comparisons between similar materials and different materials. Tensile tests have shown that our proposed infill outperforms the interlocking joint in all cases. Fractures occur even outside the interlacing area, meaning that the joint is at least as strong as the materials. The experimental results verify the enhancement of interface strength by overlapping and interlacing materials. In addition, existing computational tools have limitations in full use of material information. To the best of our knowledge, this is the first time a slicer can process overlapped material regions and create interlacing infills. The interlacing infills improve the bonding strength, making the interface no longer the weakest area. This enables MMAM to fabricate truly functional parts. In addition, the new LDMI framework has rich information on geometry and material, and it allows future research in multi-material modeling.

## 1. Introduction

Additive manufacturing (AM) technologies use a layer-by-layer stacking method to produce three-dimensional (3D) structures from input geometry. Since their introduction over 30 years ago, AM systems have become more popular in many applications. These applications have ranged from conventional products to complex design models. The geometric freedom and the freeform manufacturing technology enable the fabrication of complex structures with accurate material deposition capability at the specified positions defined in the design domain or the computer-aided design (CAD) model. Research advances in AM and the availability of new materials have unlocked a new world of endless possibilities with customization and led to complex multi-material structures [1,2]. A multi-material object is a solid model composed of varying material properties within a specified domain inside the geometry. The availability of diverse materials with unique properties is one of the main reasons for the development of multi-material structures. This allows overcoming the limitations of existing single-material AM for the development of functional objects. Therefore, multi-material additive manufacturing (MMAM) has a lot of advantages because it can fabricate advanced products that can meet today’s demand. With all the advantages of 3D printing (complex design, low cost, and customized performance, etc.), multiple materials can provide additional flexibility for desired performance and functions. For example, a customized mechanical behavior is highly desirable in tailor-made implants or soft robotics because of the requirements of varying stiffness [3,4].

Printing discrete multi-material objects is a technically difficult but economically favorable process, as it provides additional functionality and flexibility [5]. With multi-material fused filament fabrication (FFF), the fusion of materials develops the interface bond at the joint between different materials, and the strength of the bond highly depends on the compatibility between the materials. Lack of fusion is the main reason for parts breakage at the interface of materials. There are considerable research and analytical studies analyzing the effect of the interface on the overall strength of the part. Ribeiro et al. [6] developed and analyzed different interlocking joints to improve the strength of interface bonds. They showed that a macroscopic interface interlocking can improve the strength of the interface bond. Other strategies include introducing additives to induce chemical bonds and softening the material to diffuse the materials. However, these strategies still have the joint weaker than the materials themselves.

**Figure 1:**
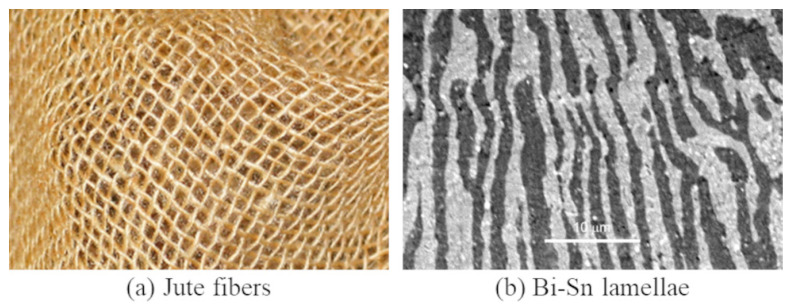
Examples of human and nature intertwining materials: (**a**) Jute fibers are interlaced to form a tenacious cloth. (**b**) The lamellar microstructures of a Bi-Sn system are composed of alternating layers of bismuth (white) and tin (black) ©2003 by Charles and Cockburn (CC BY-NC-SA 2.0 UK).

**Figure 2:**
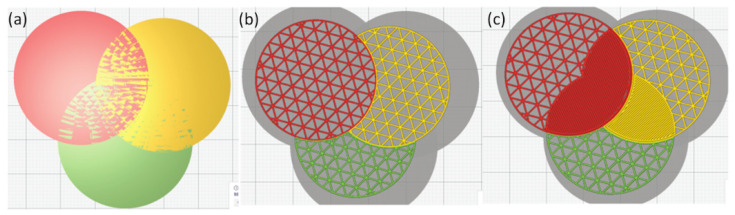
(**a**) A model having three overlapping cylinders (top view). During slicing, Cura either (**b**) lets one infill take over the overlapping region or (**c**) makes the region solid.

We make an observation that humans often intertwine materials to strengthen a product, e.g., manufacturing of composites [7] and jute fibers (Figure 1a). Interlacing fibers in clothing fabric has multiple advantages, including improved durability and resilience of a fabric [8]. Nature does the same too, such as the lamellar microstructures in eutectic alloys (Figure 1b). Many AM processes (e.g., FFF) are building and placing fibers, and the way of deposition in these manufacturing technologies has a direct effect on the mechanical properties of the fabricated part. Based on these observations on how nature and humans do to enhance mechanical properties, we hypothesize that overlapping and interlacing materials at the interface will increase the bonding strength in MMAM. To test this hypothesis, one way is to expand the interface of different materials to a small overlapping region and generate the infill within the region with both the materials. Although CAD software can overlap various parts, the generation of the interlaced infill and the toolpath needs to be done in the slicers. The capabilities of existing slicers are very limited in terms of the processing of material information in a multi-material model. For a multi-material model with overlapping geometries, these available slicers randomly assign materials to the overlapping geometry (see Figure 2).

This technology gap shows that the current computer-aided manufacturing (CAM) tools cannot make full use of material properties for MMAM. Therefore, we have to develop an advanced computational tool to process multi-material models. This paper employs a ray representation—layered depth images (LDI) [9], which is a compact format containing volumetric information. With the materials specified in the CAD model, we develop a new format named layered depth-material images (LDMI) to encode the material information in the LDI sample points. The sample points encoded with material information can tell whether a region of a layer has a single material or multiple materials. After that, we can generate interlaced infills for the multi-material regions. This paper extends our previous works [10,11], which were the first step in testing the possibility of interlacing infills. Here, we present more in-depth implementation and experiments. The contributions of this paper are:

1. Developing a new, complete LDMI framework to generate interlacing infills from a multi-material CAD model. The infills are also interlacing across layers.
2. Verifying that the interlacing also works for materials with distinct properties.
3. Finding out the required amount of overlapping to achieve the optimal bond strength.

This paper primarily focuses on the FFF technology and generates toolpaths as the result. The experimental results shown that interlacing infills is an effective approach to improve interface bond strength to be as strong as the materials, for both similar and distinct materials.

The rest of the paper is as follows. Section 2 reviews the related works; Section 3 presents the implementation details; Section 4 shows the experimental results; and Section 6 concludes the paper.

## 2. Related Work

This section reviews the related topics in MMAM, including the processes, process planning, and interface modeling.

### 2.1. Multi-Material Additive Manufacturing

There are various MMAM technologies [12], and each has its own properties. For example, Polyjet uses the ink-jetting technology to jet different materials [13], selective laser melting (SLM) melts different powders together [14], and digital light processing (DLP) changes materials by swapping the vats [15]. The FFF method [16,17] adds more materials by using more nozzles. It is probably the most accessible one because it is low cost and relatively cleaner than other methods. MMAM technologies can improve part performance by varying material compositions, which are not achievable by conventional manufacturing processes. This opens up many opportunities for design, functionality, and cost-effective high-value products [18], especially in medical and dental applications that require high performance with specific properties. However, there are still many challenges, e.g., bonding between dissimilar materials, overall low interface bonding, and multi-material data processing from CAD to CAM [12,19].

### 2.2. Process Planning for Multi-Material Printing

Today, researchers are contributing significantly to the development of CAD strategies for MMAM. One approach used to represent the multi-material model is a multi-CAD system [20]. This system is a set of CAD models with the solid information replaced by the material index. It requires users to specify the material information and the printing properties for each model separately, which adds certain complications to MMAM. There are other research works using various spatial subdivisions schemes, such as voxels [21] and surfels [22]. For example, Leung et al. [23] proposed a digital material design framework to find a proper material distribution by separating the whole domain into blocks and filling them up based on the tensor values. However, there are some issues inherent in the current AM that have halted the progress towards MMAM. The current CAM tools are still using the conventional geometry and representation [24]. Therefore, current MMAM slicers cannot process all the material information available in the CAD to the full extent [12]. A promising CAM tool is the LDI [25], which is based on ray-tracing. Compared to other slicing technologies, such as contour slicing and voxelization, ray-tracing is computationally efficient for detecting minor features in complex AM structures [26]. Besides, using graphics processing unit (GPU) can speed up the related geometrical computations by processing the pixels in parallel. Mao et al. [27] has also applied LDI to adaptive slicing and generating projection images for stereolithography (SLA) printers. Compared to the LDI, other existing methods may fail [28] or give poor performance [29] when the layer-complexity of an input model is high. Using the LDI technique can ease the challenges in multi-material AM to manufacture functional products.

### 2.3. Interface and Bonding between Materials

**Figure 3:**
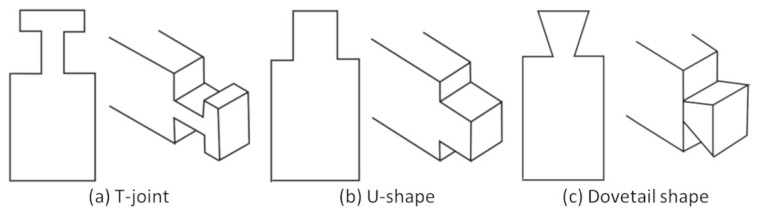
Types of Interface analyzed by Ribeiro et al. [6]: (**a**) T-shape, (**b**) U-shape, and (**c**) Dovetail shape.

Interface strength is a key concern in the property of a multi-material part. For example, the hardness at the interface differs from the materials of a multi-material part fabricated by selective laser melting [30] and there were also dimensional variations [31]. There are various techniques to develop a strong interface between materials of different compatibility. Ribeiro et al. [6] has analyzed the impact of different mechanical interlocking joints on the strength of the multi-material interface. They compared different materials and different interlocking joints (T-shape, Dovetail shape, and U-shape) for analyzing the adhesion and interlocking strength (see Figure 3). Their conclusion is that the strength of the interface joint depends much more on the macroscopic structure of the interface (mechanical interlock) than the material compatibility, and the T-shape gives the best performance. Rossing et al. [32] presented a technique to control the bonding between flexible material such as silicone and thermoplastics through mechanical interlocking. This improved the bonding strength between silicone and thermoplastics using a hybrid fabrication process. Vu et al. [33] analyzed the adhesive behavior between materials in each layer and they concluded that various printing parameters, such as print orientation, can affect the strength of the interface. Lumpe et al. [34] also showed that the interface strength highly relates to the materials and the interface design. Some structures can even have a 20% increase in strength using new multi-material designs. However, directly developing interlocking features such as T-joints and lattice structures at the interface in CAD models is a difficult and time-consuming process.

## 3. Methodology

**Figure 4:**
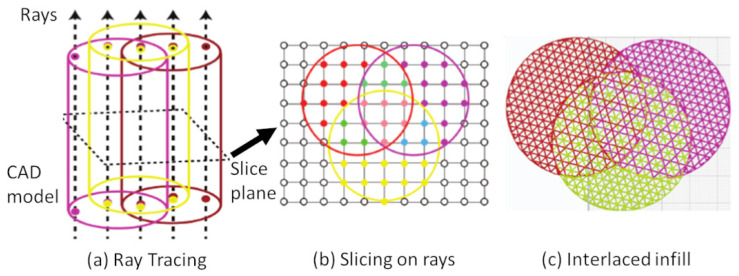
Overview of the present framework. (**a**) Tracing rays to intersect with the CAD model getting a set of material points on the rays. (**b**) Putting a slice plane to intersect with the rays to get a 2D grid with materials. (**c**) Generating interlaced infills within the multi-material regions.

Suppose an object *H* contains different material volumes, we can define it as $H=O_{1}\cup \cdots \cup O_{m}$. We can also define an overlapping region between materials *A* and *B* as $O_{A,B}=O_{A}\cap O_{B}$. The goal here is to identify the overlapping regions efficiently in the slicing process, so that we can generate the interlaced infills for these regions. As aforementioned, this paper works on top of the layered depth image (LDI) [25], which was developed based on the ray-tracing technology to encode a solid model sparsely by a set of intersection points between the rays and the model. The major difference is the inclusion of material information in the sample points, and thus we name it as layered depth-material images (LDMI). The material information is very important for the detection of different material regions in the slicing process. Figure 4 shows our proposed framework. To begin with, given the input multi-material solid model *H* (e.g., AMF file), it extracts the materials defined in *H* and stores them in a material library (*M*). It assigns each material (m∈M) with an index, so that it can retrieve them at any stage of the process. Next, assume the build direction is on the *z*-axis, it shoots a set of rays (γ∈R) from the ground along the build direction (i.e., $\hat{\gamma }=\left(0,0,1\right)$) to intersect with *H*. It stores the intersection points (s∈R∩H) together with their material index correspondingly in each ray. After that, slicing at a height is to place a plane (*P*) parallel to the ground at the height to intersect with all the rays. This results in a set of nodes (G=P∩R) forming a two-dimensional (2D) grid, and each grid node is corresponding exactly to one ray, so that it can determine the materials of the node by the corresponding ray. Finally, it groups the grid nodes into different regions according to their materials and develops the interlaced infills in the multi-material regions. We present the technical details in the following sections, starting with the ray-tracing.

### 3.1. Ray-Tracing with Material Information

LDMI represents a solid model by an image, each pixel of which records a set of sample points by shooting a ray to intersect with the model. The rays come out from the image perpendicularly and all of them are parallel to each other. Each sample point stores its coordinates, normal vectors, and material. The coordinates and the normal vectors give the geometry and the local orientation information, and the material information defines the material regions. We can place the image anywhere theoretically, but since the goal of slicing is to capture the changes in the cross-section geometry at different heights, we place the image at the ground and shoot the rays in the build direction. The image should be large enough to cover the entire model, and the image resolution uses the printing resolution of the 3D printer.

**Figure 5:**
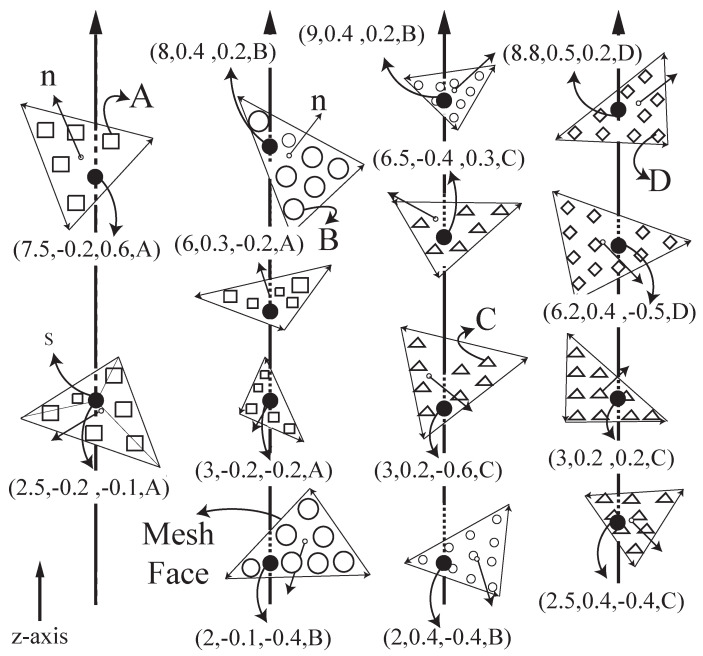
Ray tracing process to obtain the LDMI sample points.

One way to get the sample points is by computing many line-triangle intersections in the ray-tracing process as illustrated in Figure 5, but it is very time consuming. Therefore, we apply a rendering technique [25] here and extend it to include the material information. Rendering a model in the 3D space is to project and convert its triangles into a raster image at the screen. With the help of a GPU, we can use hardware acceleration to achieve real-time rendering [35]. Rasterization fills up the image pixels based on the shapes of the triangles and their properties, such as colors. If it rasters a triangle on a pixel, there must be an intersection between the triangle and the ray from the pixel. We use the color values to return the required information. Specifically, a color has four floating points for red, green, blue, and alpha channels. They can store the point’s depth value (*d*), two components of the normal vector ($n_{y},n_{z}$), and the material index (*m*). The depth value *d* is the distance from the image, which suffices to retrieve the actual coordinates because we know the ray equation, and we can calculate the third component of the normal vector by the other two: $n_{x}=1-n_{y}-n_{z}$. As a result, we can represent a LDMI sample point as ($d,n_{y},n_{z},m$) and store it on the ray of the corresponding pixel where it is located. GPU rendering significantly improves the computational performance of the ray-tracing process. For example, with the NVIDIA GeForce 920 M, generating a 1024×1024 LDMI for an input mesh of 282,624 triangular faces takes only 294 ms.

### 3.2. Slicing for Multiple Materials

After converting the CAD model to the LDMI representation, we use it in the slicing process, which divides the model as a stack of 2D layers. By intersecting a slice plane with the rays of LDMI, we can form a 2D grid at a particular height. To get all the geometry and material information in each 2D slice, there are two tasks: (1) determining the materials for each grid node, and (2) forming different material regions.

#### 3.2.1. Determining Materials for Grid Nodes

**Figure 6:**
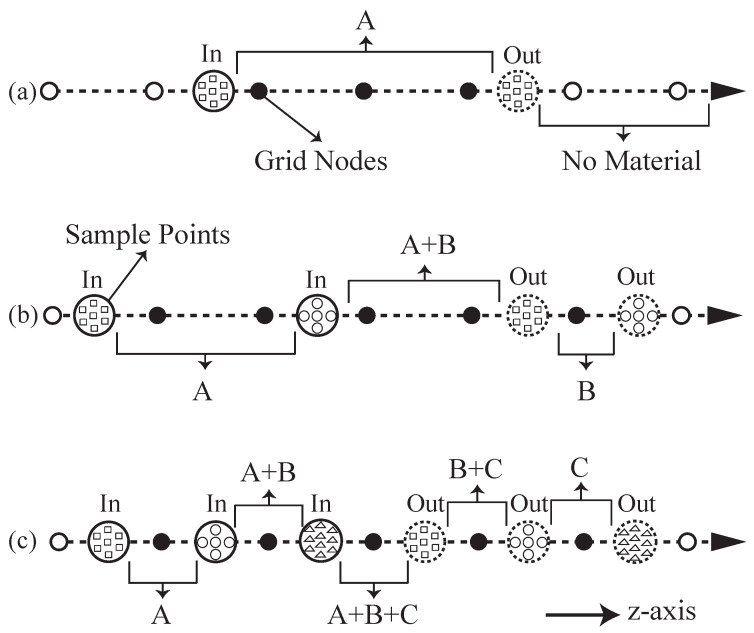
Determining materials for grid-nodes by LDMI sample points. (**a**) Single material. (**b**) Two materials. (**c**) Three materials. Note that the *z*-axis is pointing to the right.

Since the grid nodes are on the rays, the sample points of the rays can determine whether a grid node has any material and what materials it has. Figure 6 illustrates this method with three rays having various sample points. Notice that the *z*-axis is pointing to the right instead of the top in this figure, so virtually the grid nodes (dots) aligned vertically are from the same slice plane. The sample points (patterned circles) are located arbitrarily on the ray because their positions depend on the shape of the model, but the grid nodes spread uniformly along the ray since all the layers have the same height. The solid-line and the dotted-line circles indicate the In/Out of the sample points, where In means that the ray (γ) is going into the model at the sample point, and Out means coming out. We can make this In/Out classification easily by checking with the normal vector $n=(n_{x},n_{y},n_{z})$ of the sample point. It is an In point if n·γ<0, and it is Out if n·γ>0. For the case n·γ=0, the ray is a tangent line at the point on the model surface, and it is neither In nor Out, so we ignore it.

Given the In/Out classification and the materials of the sample points, we can determine the materials of the grid nodes based on where they are located among the sample points. For example, the first two grid nodes (hollow dots) in Figure 6a are located before the In-point, so they are not inside the model and should have no material. Similarly, the last two grid nodes also have no material, as they are after the Out-point. Only the nodes (solid dots) in between the In- and Out-points have materials. This applies to multiple materials too: if a node is in between the In- and Out-points of material *A* as well as the In- and Out-points of material *B*, then it contains both materials *A* and *B*, and so on (see Figure 6b,c). With the help of the LDMI sample points, the material assignment is fast, and it has a constant computational complexity: O(k), where *k* is the number of sample points on a ray which is usually small up to a few tens depending on the shape complexity of the model. It is also worthwhile to mention that, unlike other slicing algorithms, the LDMI does not have to be updated even there is a change in the number or the height of layers. No matter using how many grid nodes or how redistributing them, the sample points are unaffected. This provides LDMI with an advantage to achieve fine quality prints without extra computational costs.

#### 3.2.2. Forming Material Regions

**Figure 7:**
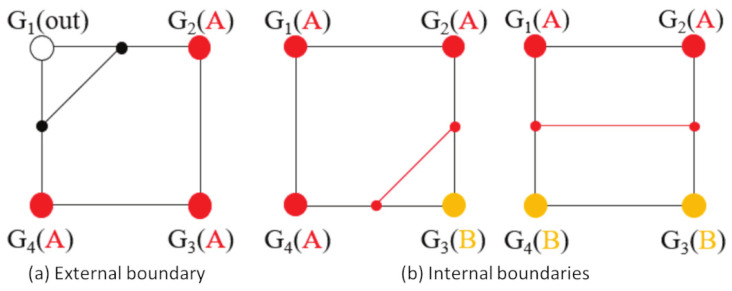
Development of contour lines in cells with ‘G’ representing grid-nodes on a slicing plane. (**a**) External boundary, (**b**) Internal boundaries.

After assigning all the grid nodes of a slice plane with materials, the 2D grid essentially is a discrete material field. The next step is to extract clear boundaries to bound different material regions, so that we can plan the fabrication of each region. The boundaries are important in the interface modeling and in the development of interlaced infill within the multi-material regions. Here, we apply the marching squares algorithm to the generation of contours for the 2D material field. Specifically, the algorithm processes four neighbor nodes forming a cell at a time and determines contour lines as part of the contours that pass through this cell. It then fuses the individual lines into the entire contours. The construction of contour lines depends on the cell configuration, i.e., the material combination. Figure 7 shows some configurations and the output lines for the cells. In short, it places a line between the nodes that have different materials. If all the nodes in a cell have no material or the same material, there are no lines in the cell. For a line that is placed between a node with no material and a node with a material (Figure 7a), it is a part of the external boundary, i.e., the model surface. For a line that is placed between different materials (Figure 7b), it is a part of the internal boundary. After processing all the cells and completing the contours, it applies a linear interpolation along the contours to make them smooth.

### 3.3. Generating Interlaced Infills

Given the material interfaces and regions defined in a 2D plane, this section presents the approach to generate the interlaced infill. The present method can generate the interlaced infills on the fly in the CAM phase, without altering the CAD model. If a region has material, it can have a single material or multiple materials. A single-material region uses a normal infill, and we generate an interlaced infill for the multi-material regions. Without loss of generality, this paper uses triangular infills to explain the generation of interlaced infills, but we can use other types of infills similarly. A triangular infill has three sets of parallel lines inclined at angles of $0^{\circ }$, $60^{\circ }$, and $120^{\circ }$. It places the lines uniformly separated by a distance Δ, which is determined by the desired infill ratio *I*, i.e., the larger the infill ratio, the smaller the line distance. To develop an interlaced infill in the multi-material regions, we divide the overall infill ratio *I* by the number of materials in the region. For example, if a region has *n* materials, the infill ratio for each material is $I_{m}=I/n$, and the line distance is $\Delta_{m}=n\Delta$. The infill lines of different materials should stagger in the region, so we introduce an infill shift (*s*), which is based on the line distance and applied separately to each material, i.e., $s_{1,\cdots ,n}=\left\{0,\Delta ,\cdots ,\left(n-1\right)\Delta \right\}$.

**Figure 8:**
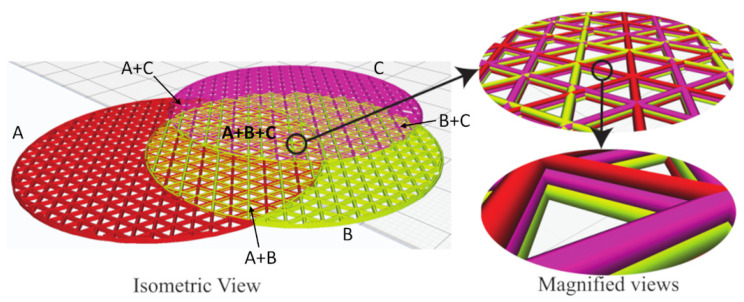
Visualization of interlaced infills on a model of three overlapping cylinders with materials *A*, *B*, and *C*.

Figure 8 shows an example of three overlapping cylinders with materials: *A*, *B*, and *C*. There are seven different material regions, including three single-material regions, three double-material regions (A+B, B+C, A+C), and one triple-material region (A+B+C). The overall infill ratio is I=20% and thus the line distance is Δ=2.5 mm. In the single-material regions, the infill shift is s=0, as there is no need to shift the infill lines. In the double-material regions (e.g., A+B), the whole infill comprises the two materials. Each of them occupies half of the infill volume, and thus the infill ratio for the materials is $I_{A}=I_{B}=10\%$, leading to their line distances of $\Delta_{A}=\Delta_{B}=5$ mm. To alternate the materials, we shift one material by the line distance Δ, i.e., $s_{A}=0$ mm and $s_{B}=2.5$ mm. Similarly, in the triple-material region (A+B+C), $I_{A}=I_{B}=I_{C}=6.67\%$, $\Delta_{A}=\Delta_{B}=\Delta_{C}=7.5$ mm, $s_{A}=0$ mm, $s_{B}=2.5$ mm, and $s_{C}=5$ mm. To further enhance the interlacing effect among layers, we also apply the infill shifting to the materials alternatively in subsequent layers (see the magnified views in Figure 8). For example, after printing layer 1 as aforementioned, layer 2 uses $s_{A}=2.5$ mm, $s_{B}=5$ mm, and $s_{C}=0$ mm, so that the order is A→B→C in layer 1, then C→A→B in layer 2, and so on. This can interlace the materials not only in 2D but also uniformly throughout the entire model.

This example has 150 layers, and the entire process from the sampling to the G-code generation takes only a few seconds. The ray-tracing, with the help of GPU (NVIDIA GeForce 920 M), takes 421 ms to build a 2048×2048 LDMI, i.e., 4.2 M rays. The other sub-processes run in a CPU (Intel Core-i5 7th Generation) with 8 GB RAM. The slicing takes 183 ms to classify materials on the grid nodes and 524 ms to form the material regions. The time for generating the G-codes is 3678 ms, which is a common step in any slicing process.

## 4. Results

**Figure 9:**
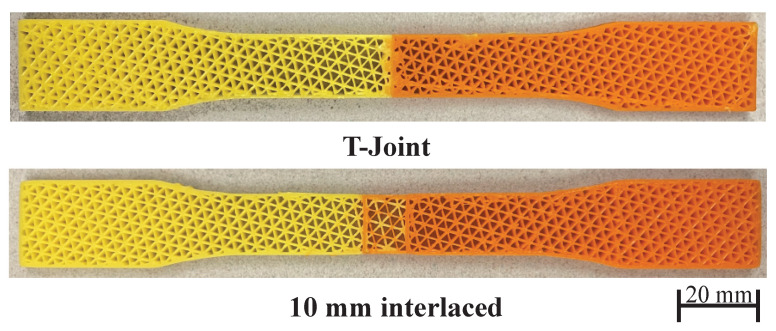
The dog-bone samples for tensile test.

**Figure 10:**
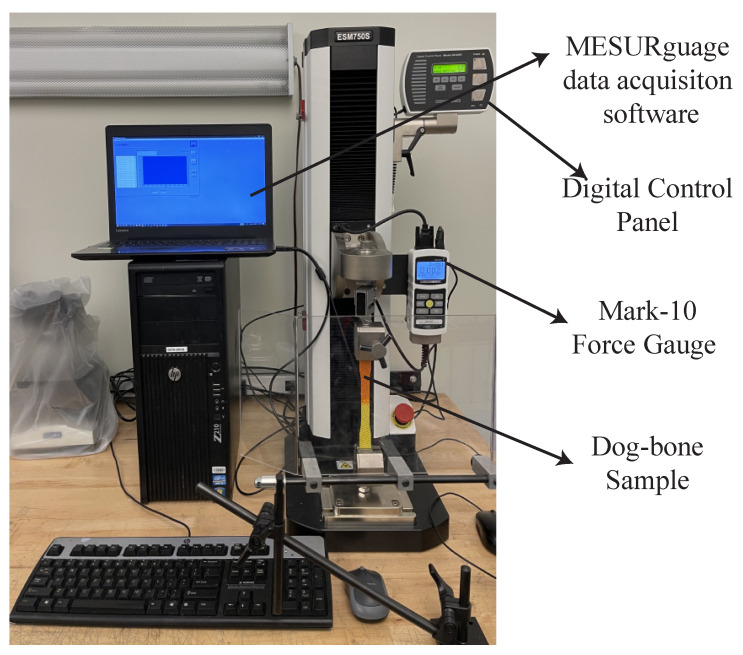
The experimental setup for tensile test.

With the developed LDMI framework, now we can model the multi-material interface, and this section tests our hypothesis that interlacing infills increases interface strength. To measure the interface strength, we use the ASTM-D638 standard to fabricate a dog-bone shape with two materials touching at the middle of the dog-bone, as shown in Figure 9. The overall infill ratio is 40%, and we 3D print the samples with an Ultimaker 3. We have weighted the samples to make sure that they have the same amount of material. Then, we stretch the samples to their breaking point with a tensile tester—Mark-10 ESM750SLC (see Figure 10) and get the stress-stain data for the analysis.

**Figure 11:**
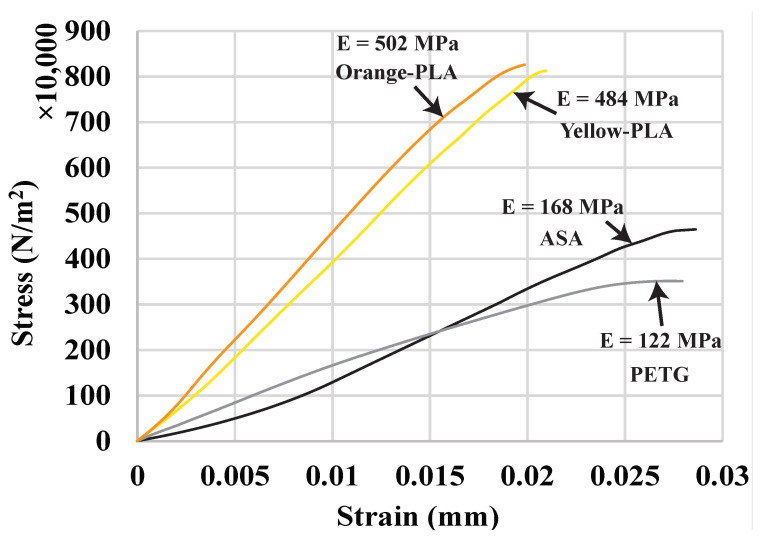
Tensile test curve for single-material samples.

There are three sets of experiments. The first experiment uses the same material (PLA) but different colors (orange and yellow) to compare the interface strength among various joint configurations. The second experiment further verifies the effectiveness of the interlaced infills in different materials (PLA, PETG, and ASA). The last experiment tries to find out if there is a relationship between the interface strength and the overlap size. We have tested all the configurations three times and averaged the values (±0.1 MPa). As a reference, we have also tested all the materials used in the experiments individually with the same dog-bone shape and reported their data are in Figure 11 and Table 1, including the Young’s modulus (*E*) and the ultimate tensile stress (UTS). Apparently, the PLA is much stiffer than the other two materials and can carry at least two times more loads before fracture, but it fractures at around 0.02% strain, while PETG and ASA fracture at around 0.028% strain.

### 4.1. Joint Configurations

To compare the bonding strength of different interfaces, we have tested three joint configurations: (1) a butt joint, (2) the interlocking T-joint, and (3) the proposed overlapping joint with interlaced infill. The butt joint joins the materials by simply placing them closely next to each other with no special shaping. The interface strength for this joint purely depends on the material compatibility and the adhesion between the material surfaces introduced in the 3D printing process. It is the baseline to show the improvement achieved by other configurations. The dimension of the T-joint is as follows. The width and height of its flange (horizontal) are 2.10 mm and 1.35 mm, and the width and height of its web (vertical) are 0.85 mm and 0.62 mm. There are in total five T-joints at the interface, three on one side and two on the other. The interlaced infill has an overlap size of 10 mm. To make the effect of joints prominent, this experiment tests similar materials (orange PLA and yellow PLA).

**Figure 12:**
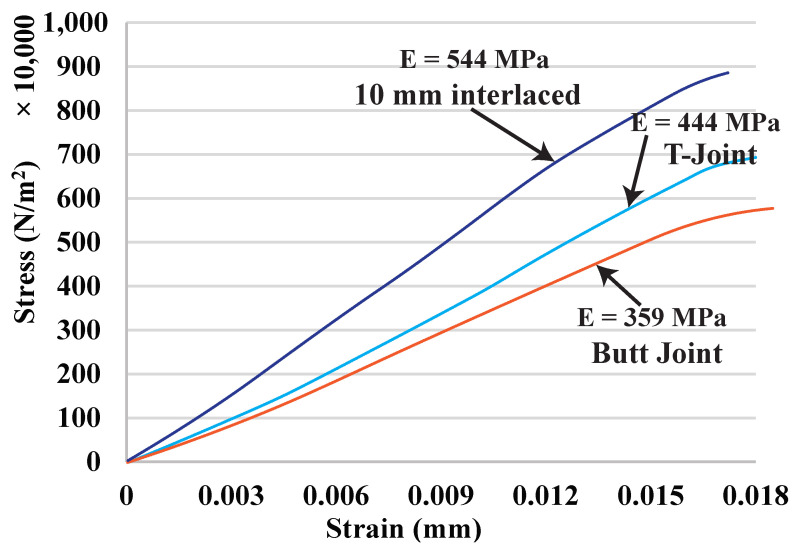
Tensile test curves for different joint configurations.

**Figure 13:**
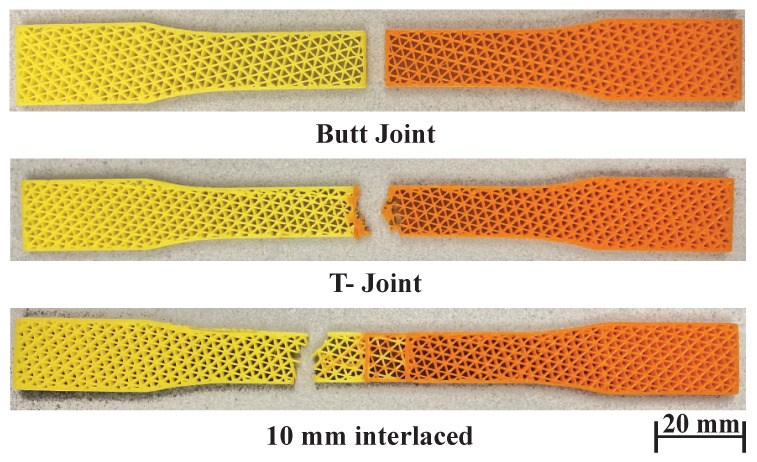
Fractured samples of butt joint, T-joint, and interlaced infill.

Figure 12 and Figure 13 and Table 2, show the testing results and the fractured samples. All have a similar fracture strain of around 0.018%. For the butt joint configuration, the observed *E* value is 359 MPa, and the observed UTS is 5.79 MPa, which are far less than the strength of the PLA materials themselves—*E* is around 500 MPa and UTS is around 8.7 MPa (see Figure 11 and Table 1). The tested sample shows that the fracture happens exactly at the joint. This reveals that the weakest location of a multi-material structure is indeed at the interface, and the amount of reduced strength is significant (∼40% reduction). The T-joint introduces a mechanical interlock effect besides the adhesion. Our testing results are in line with the finding in the previous work [6] that the T-joint can improve the interface strength. There is a 20% increase in UTS compared to the butt joint (6.95 vs. 5.79 MPa). However, it is still 20% weaker than the PLA materials, and the fracture also happens at the joint. The proposed interlaced infill shows a very promising result. The sample actually fractured at a distance of 12 mm away from the joint area, meaning that the joint is at least as strong as the materials. This is also evidenced by the observed E=544 MPa and UTS =8.88 MPa, which is not only a 53% improvement compared with the butt joint, but also as strong as the PLA materials. The results prove that overlapping and interlacing materials can increase the interface strength between similar materials.

### 4.2. Materials with Distinct Properties

**Figure 14:**
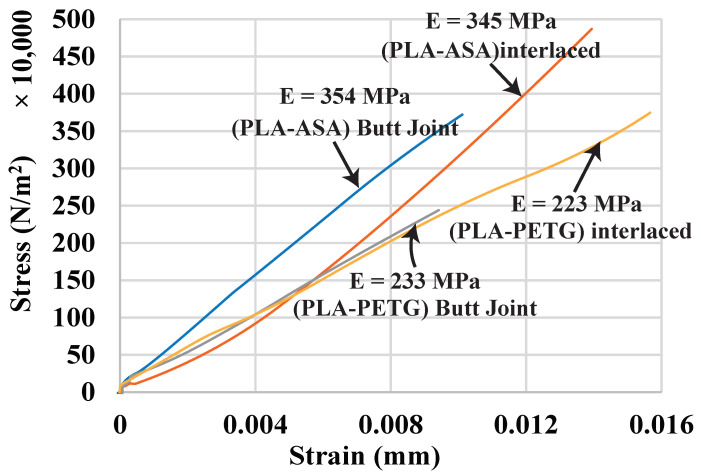
Tensile test curves for different material combinations and joint configurations.

**Figure 15:**
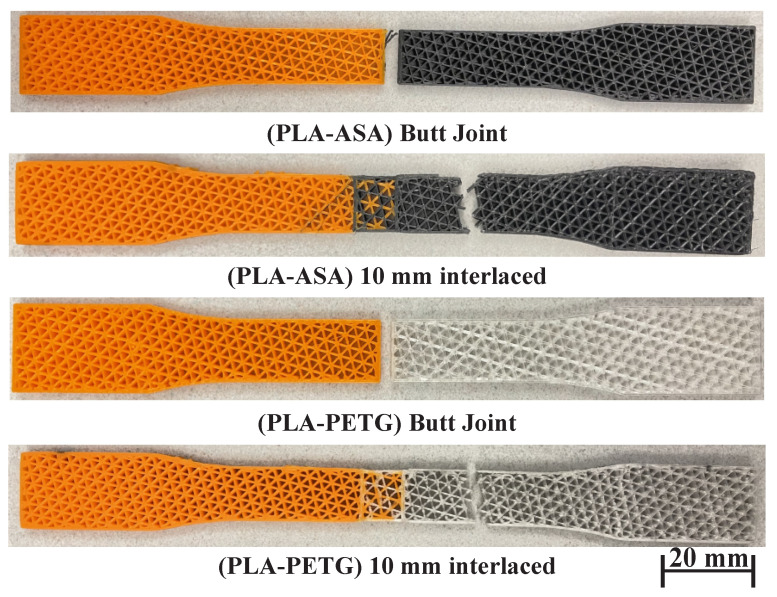
Fractured samples of butt joints and interlaced infills for the material combinations: PLA-ASA and PLA-PETG.

The previous experiment uses two very similar materials, so this experiment tests the interlaced infill on some distinct materials to further confirm its capability. There are three materials and two combinations: PLA-ASA and PLA-PETG. It tests each combination with both the butt joint and the interlaced infill, and the overlap size for the interlaced infill is also 10 mm in this experiment. Figure 14 and Figure 15, and Table 3, show the testing results and the fractured samples. Both material combinations show the same trend as the results of the experiment using similar materials: the interface strength of the butt joint is lower than the weak material in the combination. For example, the UTS of the material ASA is 4.85 MPa, but the UTS of the combination PLA-ASA is only 3.58 MPa, in addition to the butt joint fractures at the interface. In contrast, the samples of interlaced infill fracture inside the weak material at a distance away from the joint. They also have the UTS of the weak material, having an improvement of 34% and 57% for PLA-ASA and PLA-PETG, respectively, compared with the butt joint. This reveals that the interface is even stronger than the weak material. In addition, for both PLA-ASA and PLA-PETG, the butt joint fractures much earlier at around 0.009% strain, while the interlaced infill fractures at around 0.015% strain.

### 4.3. Overlap Size and Interface Strength

**Figure 16:**
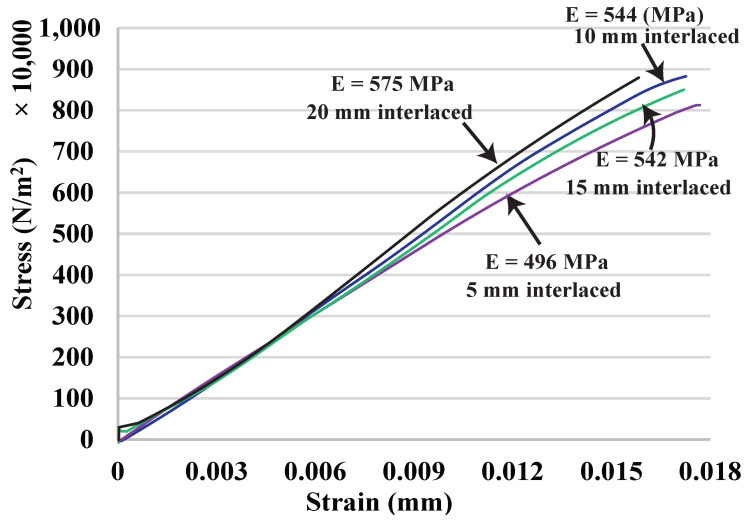
Tensile test curves for different sizes of overlap.

**Figure 17:**
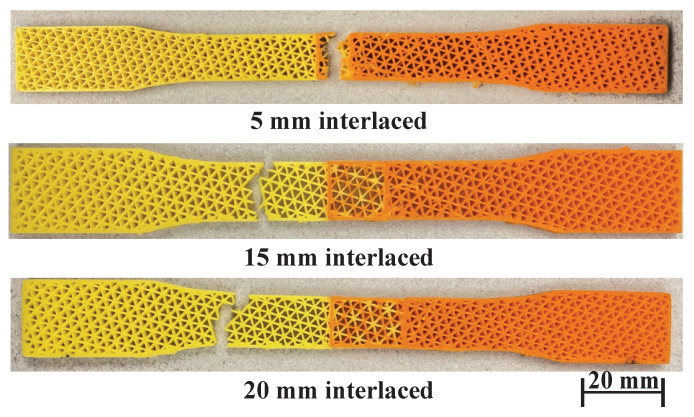
Fractured samples of interlaced infills with different overlaps: 5 mm, 15 mm, and 20 mm.

After knowing that interlacing infills is effective, this experiment wants to find out how critical the size of overlap is to the interface strength and what their relationship is. The experiment is as follows. First, it uses the materials with similar property (orange PLA and yellow PLA) to better observe the changes because of the joint area. Second, it tests four different sizes of overlap: 5 mm, 10 mm, 15 mm, and 20 mm. Figure 16 and Figure 17, and Table 4 show the testing results and the fractured samples (the 10 mm one is in Figure 13). Although they have a similar fracture strain of around 0.016%, the data shows that as the size of overlap increases from 5 mm to 10 mm, the interface strength in terms of UTS is also higher from 8.25 MPa to 8.88 MPa. We can also see that the sample of 5 mm overlap fractures in the joint area, so that the joint is indeed weaker than the PLA materials (∼8.7 MPa), but the difference is not large, and it is still better than the T-joint (6.95 MPa). The 10 mm, 15 mm, and 20 mm samples all fracture outside the joint, and they have similar UTS (8.77–8.88 MPa). This reveals that it has already maximized the performance at the overlap size of 10 mm, and the results are consistent beyond this size. Therefore, the overlap size can be between 5 and 10 mm, which can already provide a better property than mechanical interlocking joints.

## 5. Discussion

One major concern of MMAM is the strength at the interface between materials. Particularly, FFF is well-known to have low diffusion between material strands and weak interlayer bonding [36]. Observing how human and nature intertwine materials to strengthen various parts, we made a hypothesis of interlacing infills to enhance the multi-material interface strength. To test the hypothesis, we have created the LDMI tool to construct the interlaced infills and conducted several experiments to generate and gather data. The experimental results show that the interlaced infills indeed perform better than other mechanical interlocking joints and thus verify our hypothesis. The improved interface property is because of the increased contact area between the materials by interlacing. As the extrusion of materials extends from the single-material regions to the multi-material region, greater contact area means greater frictional force between the materials. The situation is like the inseparable books experiment, where even two people cannot pull apart two interleaved books, and this immense force comes from the small frictional forces between every interleaved pages. In addition, the huge frictional forces at the interface hold the materials strongly together, result in a blended property. This is like the fiber reinforcement of a polymer [37], which disperses the stiff fibers throughout the relatively weak polymer to enhance the property of the polymer. Here, the interface is no longer the weakest area because of reinforcement, and thus the fracture happens outside the joint area. The present method has a potential for material reinforcement by interlacing a stronger material with a weaker one.

## 6. Conclusions and Future Work

Based on the observation that nature often intertwines materials to increase strength, this paper hypothesizes that overlapping and interlacing infills of different materials enhances the interface strength between the materials of a multi-material part. However, existing slicers cannot generate the interlaced infill. To test the hypothesis, this paper also develops a novel slicer—named layered depth-material images (LDMI)—for multi-material additive manufacturing (MMAM) to process the material information efficiently. The LDMI framework takes a multi-material computer-aided design (CAD) model as an input and applies rendering techniques to encode the material data into the sample points of the model. It then uses the encoded data in the slicing process to identify different material regions in each slice plane and generates the interlaced infills in the multi-material regions.

We conducted experiments to compare different joint configurations (butt joint, T-joint, and interlaced infill) and different material combinations (PLA-PLA, PLA-ASA, and PLA-PETG). The tensile test results show that the T-joints are better than the butt joints, and the interlaced infills outperform both. The samples of interlaced infill even fracture outside the joint area, meaning that the interlacing leads to a joint at least as strong as the materials. The trend is the same for different material combinations. This verifies the hypothesis, and the approach is promising to create strong multi-material parts. The method can also apply to the reinforcement of a weaker material by interlacing it with a stronger material. We also found that an overlap size of 05-10 mm suffices to have the full interlacing impact on the interface.

The major limitations of the present work include being only tested with the triangular infills, the dog-bone shapes, and the fused filament fabrication (FFF) process. Triangular infill is normally the strongest infill pattern because triangles are least likely to deform. It will be interesting to find out whether the interlacing has the same impact on other infill patterns as well. We also plan to extend the present work to functionally graded infills by varying the infill ratio of each individual material, which will require a new way to interlace the materials to achieve the desire mixing ratio. In addition, we will test more complex shapes and loading conditions to further confirm the method’s capability. Finally, will will also test on other MMAM process such as MultiJet printing (MJP) to validate its generality.

## Figures and Tables

**Table 1:** Tensile test data for single-material samples.

Materials	*E* (MPa)	UTS (MPa)
Orange PLA	502	8.74
Yellow PLA	484	8.68
ASA	168	4.85
PETG	122	3.69

**Table 2:** Tensile test data for different joint configurations.

Joints	*E* (MPa)	UTS (MPa)	Improve
Butt	359	5.79	-
T-joint	444	6.95	20%
Interlaced	544	8.88	53%

**Table 3:** Tensile test data for different material combinations and joint configurations. The unit for *E* and UTS is MPa.

Materials	Joints	*E*	UTS	Improve
PLA-ASA	Butt	354	3.58	-
	Interlaced	345	4.79	34%
PLA-PETG	Butt	233	2.38	-
	Interlaced	223	3.74	57%

**Table 4:** Tensile test data for different sizes of overlap.

Overlaps	*E* (MPa)	UTS (MPa)
5 mm	496	8.23
10 mm	544	8.88
15 mm	542	8.74
20 mm	575	8.86

## Data Availability

This paper comes and extends from a graduation thesis [11].
